# Optimization of Processing Parameter and Mechanical Response Analysis of Advanced Heterogeneous Laminated Composites Using Ni/Al Foils by In Situ Reaction Synthesis

**DOI:** 10.3390/ma15248892

**Published:** 2022-12-13

**Authors:** Ying Sun, Shijian Yuan

**Affiliations:** 1 Institute of Precision Forming for High Performance, Dalian University of Technology, Dalian 116024, China; 2 National Key Lab for Precision Hot Processing of Metals, Harbin Institute of Technology, Harbin 150001, China

**Keywords:** heterogeneous laminated composites, parameter optimization, intermetallics, microstructure, mechanical properties

## Abstract

The advanced heterogeneous laminated composites were successfully fabricated by vacuum hot pressing using Ni and Al foils by in situ solid-state reaction synthesis. The effects of holding time and temperature on the microstructure and phase distribution were analyzed using scanning electron microscopy. Based on the optimized processing parameters, the microstructure and phase transformation, and the relationship between the microstructure and the corresponding mechanical properties were discussed in detail. To clarify the mechanical response of the laminated structure, the deformation microstructure and fracture characteristics were studied by scanning electron microscopy and electron backscatter diffraction. The results indicated that the evolution of the interfacial phases in the laminated composite occurred via the sequence: NiAl_3_, Ni_2_Al_3_, NiAl, and Ni_3_Al. An interface between the Ni and Ni_3_Al layers without cracks and voids formed due to the uniform pressure applied during hot pressing. The laminated composites hot pressed under 620 °C/5 MPa/1 h + 1150 °C/10 MPa/2 h exhibited the best ultimate tensile strength of 965 MPa and an elongation of 22.6% at room temperature. Extending the holding time during the second stage of the reaction synthesis decreased the thickness of the Ni_3_Al layer. This decreased the tensile strength of the laminated composite at 1000 °C but improved the tensile strength at room temperature. Moreover, the layer–thickness relationship of the laminated structure and the matching pattern were important factors affecting the strength and elongation of the laminated composites. The reinforcement form of the materials was not limited to a lamellar structure but could be combined with different forms of reinforcement to achieve continuous reinforcement over a wide range of temperatures.

## 1. Introduction

Ni_3_Al has attracted great attention for high-temperature structures in aerospace applications due to its excellent mechanical properties at elevated temperatures, including its high melting point, low density, and oxidation resistance [1]. However, the intrinsic brittleness of Ni_3_Al alloy leads to its poor ductility and fracture toughness. Metal–intermetallic composites offer an attractive combination of physical and mechanical properties, such as a high strength, high toughness, and low density [2]. The mechanical properties of laminated composites can be optimized by group element materials, group element layer thickness, interface thickness, and interface configuration. This ensures that metal–intermetallic composites can maintain the high strength and stiffness of intermetallic compounds while retaining the high toughness and ductility of metals. The metal–intermetallic composites are attractive in a wide range of engineering applications. Because the tailored mechanical properties can be obtained by controlling processing parameters, the composites are suitable for a service environment at high temperatures and also meet the requirements of the assembly conditions at ambient temperatures. The applications include compressor vanes and cylinder heads of automotive engines, as well as high-performance aerospace applications [2,3,4]. Therefore, the design and manufacturing of laminated metal–intermetallic compound composites by introducing microstructural heterogeneity can realize useful intermetallic compounds.

Rawers et al. [5] and Alman et al. [6] demonstrated a near-net shaping method that can be used to prepare metal–intermetallic composites via in situ reaction synthesis between constituent Ni/Al foil elements. Because the starting material was metal foils, this approach was a reasonably inexpensive process. Compared with pre-alloyed intermetallic foils, this method can control the distribution of phases and protect the interfaces between layers. Compared to Ni and Al powder sintering or NiAl_3_ spray deposition, the solid-state reactive synthesis method by the hot pressing of Ni and Al foils can approach large-scale industrial production for the fabricating of thin sheets and thin-walled components at low cost. Moreover, it is possible to achieve the metal–intermetallic laminated composite with low oxygen contents by the synthetic approach compared to the sintering of standard aluminum and nickel powders. Reaction synthesis is an essential bridge between the starting foils and the Ni-Al metal–intermetallic laminated composites, which involves the self-propagating high-temperature synthesis mode and the thermal explosion mode [7]. Many researchers [8,9,10,11] have studied the phase transition and the reaction products at different reaction stages by using Ni/Al foil diffusion couples. Kim et al. [7] investigated the reaction synthesis of a NiAl/Ni microlaminated composite fabricated by alternatively stacked Ni and Al foils. The reaction products were converted to NiAl and Ni_3_Al at the temperature of 900–950 °C and the applied pressure of 50–100 MPa, with the initial thickness ratio (Ni:Al) of 1:1. Seyring and Rettenmayr [12] studied the formation of the Ni_3_Al phase at the Ni/NiAl interface and established a specific crystallographic relationship between the Ni_3_Al phase and the neighboring Ni/NiAl interface, which was evaluated using EBSD and STEM-EDX. As reported by Wu et al. [13], the microstructure and comprehensive properties of Ni_3_Al-based alloys were greatly affected by heat treatments. As investigated by Wu et al. [14], the microstructure evolution and mechanical properties affect, at room temperature or high temperature, the multiphase Ni_3_Al-based alloy during the laser remelting treatment. Ogneva et al. [15] investigated the microstructure and phase transitions of Ni-Al multilayer composites obtained by spark plasma sintering with Ni and Al foils. During the sintering process, the Ni_2_Al_3_, NiAl, and Ni_3_Al layers were formed in the composites under the condition of 900–1100 °C and the pressure of 10–30 MPa. However, the reaction products were studied based on certain processing parameters. Despite the similarity of the phase constituents of the composites, the processes of phase formation and disappearance of the lamellar structure and the role of process parameters in controlling the thickness of each layer have not been investigated sufficiently.

Konieczny [16] studied the tensile properties of the laminated Ni/Ni_2_Al_3_/NiAl_3_ and Ni/Ni_3_Al/NiAl composites fabricated by reaction synthesis using 0.4 mm Ni foils and 0.15 mm Al foils under the conditions of 620 °C/2 h/1 MPa and 1150 °C/4 h/1 MPa. Mizuuchi et al. [17] studied the microstructure and mechanical properties of Ni-aluminide-reinforced Ni matrix composites fabricated by 0.05 mm Ni/0.012 mm Al foils and 0.05 mm Ni/0.024 mm Al foils by pulsed current hot pressing equipment. Kim et al. [18] investigated the tensile and fracture properties of NiAl/Ni microlaminated composites prepared by reaction synthesis. Ogneva et al. [15] studied the effect of sintering temperature and pressure on the microstructure and mechanical properties of Ni-Al metal–intermetallic laminated composites. However, optimization of processing parameters was more important for providing desirable mechanical properties. In addition, the relevance of the high ductility of the Ni matrix in providing desirable mechanical properties for Ni-Al metal–intermetallic laminated composites has not been fully investigated. Wang et al. [19] studied the effect of Ni foil thickness on the microstructure and tensile properties of reaction synthesized Ni/Ni_3_Al multilayer composites. Xi [20] studied the microstructure and mechanical properties of reaction synthesized Ni-Al intermetallic sheets by different thickness ratios of Ni and Al foils under the conditions of 640 °C/20 MPa/1 h + 1200 °C/20 MPa/1 h. However, the process parameters have an influence on the mechanical properties of the laminated materials, which are controlled essentially by adjusting the phase distribution and phase components to control the mechanical properties. It is difficult to obtain the desired mechanical properties by changing the thickness ratios of Ni and Al foils under a certain process parameter. Overall, the influences of the hot pressing temperature and holding time on the microstructures and phase composition have not been sufficiently researched. It is essential to clarify the microstructure and mechanical properties of the advanced laminated composites under the optimized processing parameters to provide guidance for practical industrial applications.

In this study, the advanced heterogeneous laminated composites were prepared via hot pressing at various temperatures and holding times to investigate the effect on the microstructure and mechanical properties. The microstructures and phase distribution of the laminated composites were characterized in detail. Moreover, the mechanical properties were measured to establish structure–processing property relationships to provide a new strategy for manufacturing Ni/Ni_3_Al thin-walled components.

## 2. Experimental Procedure

### 2.1. Materials and Fabrication Processes

**Figure 1:**
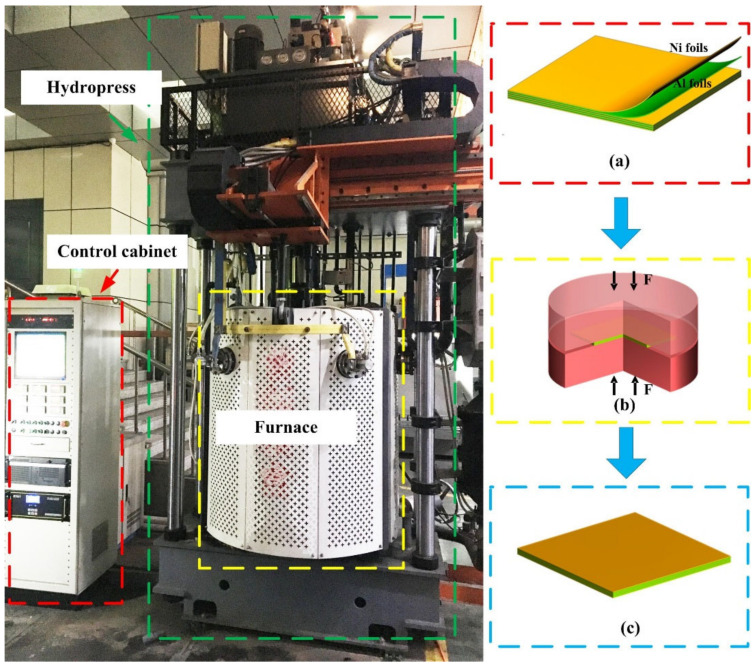
Schematic of the preparation procedure for the laminated composites. (**a**) Stacked alternately Ni and Al foils; (**b**) hot pressing to obtain the laminated composites; (**c**) the final laminated composites.

Commercial pure Ni foils (99.93% wt.% pure) and Al foils (99.99% wt.% pure) were used as the raw materials for fabricating the laminated composites. Pure Ni foils and pure Al foils were cut into 100 mm × 100 mm squares and treated in an ultrasonic acetone bath for 30 min. The Ni foils (19 pieces) with a thickness of 0.06 mm and Al foils (18 pieces) with a thickness of 0.02 mm were alternately stacked to prepare sandwich structure with a sequence of Ni/Al/Ni/Al……Ni/Al/Ni. The Ni foils were placed in the topmost and lowest positions of the Ni/Al composited structure. Figure 1 shows the schematics of the preparation procedure for the laminated composites. The stacking laminates Ni/Al foils of Ni and Al were prepared and then hot pressed in a graphite mold in a vacuum hot pressing furnace. The stacked laminates Ni/Al foils were first preheated at 620 °C for 1 h and then heated to 1100–1200 °C with a holding time of 0.5–2 h under a pressure of 10 MPa and then cooled to room temperature in the furnace vacuum chamber. The working vacuum degree of the furnace was 5 × 10^−3^ Pa, and the pressure control accuracy of the hydropress was ±5%. Table 1 summarizes the hot pressing parameters and their corresponding marks for the convenience of expression and presentation.

### 2.2. Materials Characterization

**Figure 2:**
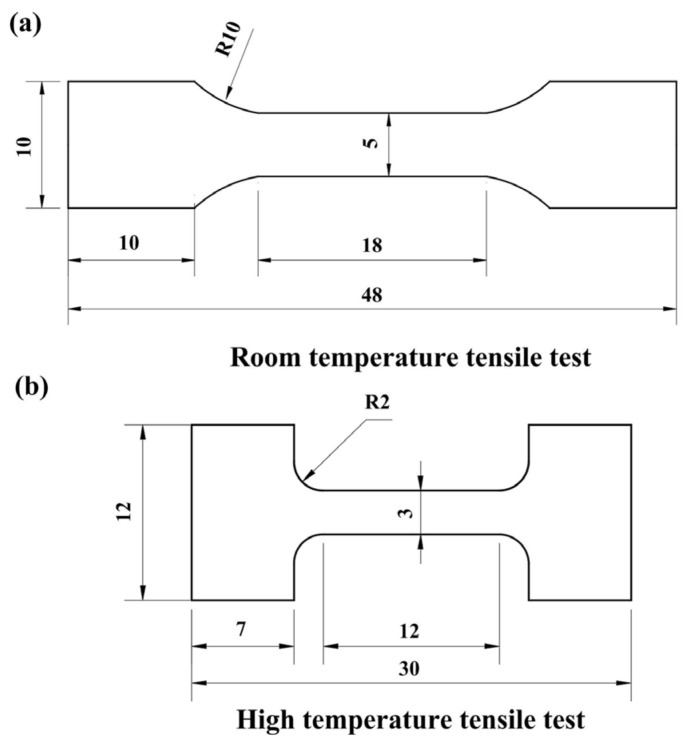
Dimensions of the specimens used for tensile testing. (**a**) Room-temperature tensile test; (**b**) high-temperature tensile test (all dimensions are in mm).

A scanning electron microscope (SEM, ZEISS SUPRA 55) equipped with energy-dispersive spectroscopy (EDS) was used for microstructure observation, chemical composition measurement, and fracture surface analysis. The specimens for BSE and EBSD were ground and then subjected to electro-chemical polishing with a solution of 6% perchloric acid, 35% butarol, and 59% carbinol (vol%) at −30 °C. The polishing voltage and current density were 25 V and 0.6 A, respectively. The geometrically necessary dislocation (GNDs) maps were calculated from the EBSD data by Channel 5 analysis software (Version 5.0.9.0). The EBSD data were collected with a step size of 0.5 μm. Uniaxial tensile tests were performed using a Shimadzu AG-X Plus instrument with an initial strain rate of 1 × 10^−3^ s^−1^ at room temperature. High-temperature tensile tests were conducted on an electronic universal material testing machine (Shimadzu AG-X Plus, Kyoto, Japan) with a strain rate of 1 × 10^−3^ s^−1^ at 700–1000 °C. The detailed specimen geometry is shown in Figure 2. The tensile specimens were cut by electrical discharge machining. Before testing, the tensile specimens were mechanically ground and subsequently polished with a diamond suspension containing 0.5 µm particles. Strain gauges were attached to the specimens to measure tensile strain, and the tensile tests were repeated three times to guarantee repeatability. Each sample was maintained at the designated temperature for 5 min to obtain a uniform deformation temperature. After deformation, the samples for high-temperature tensile tests were quickly removed from the resistance furnace and quenched in water.

## 3. Results

### 3.1. Microstructural Analysis of the Laminated Composites

**Figure 3:**
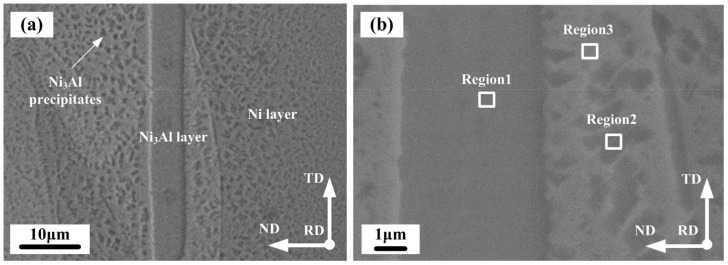
Low-magnification SEM image (**a**) and high-magnification SEM image (**b**) of the laminated composites after hot pressing under 620 °C/5 MPa/1 h + 1150 °C/10 MPa/2 h. RD, ND, and TD are the rolling, normal, and transverse directions, respectively.

Figure 3 shows the cross-sectional microstructure of the laminated composites after hot pressing under 620 °C/5 MPa/1 h + 1150 °C/10 MPa/2 h, in which a uniform multilayered structure with straight bonding can be clearly observed. The element distributions are displayed in the high-magnification SEM image in Figure 3b. The EDS point scanning results are listed in Table 2. As indicated in Figure 3, the microstructure mainly consisted of Ni_3_Al layers (dark gray region) and Ni layers (dark white region), as detected by SEM-EDS. The original Al layers were not detected, indicating that the Al foils were alloyed entirely, leading to the formation of a reaction synthesis region, which was visible as a dark gray region. Sound interfacial bonding was obtained by the interdiffusion of Ni and Al foils in the normal direction. The uniform thickness of the original foils contributed to the formation of a straight bond between each layer. Region 1 was detected as the Ni_3_Al layer. Regions 2 and 3 were detected as Ni_3_Al precipitates and the Ni matrix, respectively. These three regions constituted a dual-phase laminated structure. The structure was composed of Ni matrix layers and Ni_3_Al layers that were parallel to each other. There were almost no microvoids or cracks observed in the middle of the Ni and Ni_3_Al layers or their interface due to the uniform pressure applied during hot pressing bonding. This indicated that a well-bonded laminated composite was achieved.

### 3.2. Structural Design and Fabrication Processing Parameter Optimization

**Figure 4:**
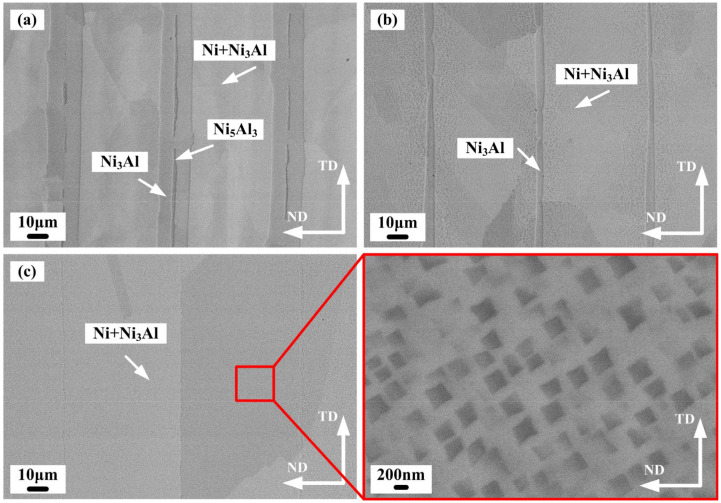
BSE images of the laminated composites. (**a**) N1; (**b**) N2; (**c**) N3.

A laminated metal–intermetallic composite was designed based on the Ni-Al material system. The fabrication process of the laminated composite strongly depended on the interdiffusion between Ni and Al atoms. Therefore, the microstructure and mechanical properties were highly dependent on the temperature and holding time during hot pressing. Figure 4 depicts the microstructure of samples N1, N2, and N3 fabricated under different processing parameters. As shown in Figure 4a, the microstructure mainly consisted of three phases: Ni, Ni_3_Al, and Ni_5_Al_3_. Thin Ni_5_Al_3_ layers were observed between Ni_3_Al layers, where the Ni_5_Al_3_ layers presented a discontinuous pattern in the Ni_3_Al layers. The Ni_5_Al_3_ phase can nucleate above 600 °C, and the formation of the Ni/Ni_3_Al/Ni_5_Al_3_ lamellar structure in Figure 4a was a result of the Ni_5_Al_3_ phase not completely changing into the equilibrium mixture of the Ni_3_Al + NiAl phases above 700 °C [21]. Microvoids were not observed in the middle of the Ni_5_Al_3_ layers. The average thicknesses of the Ni_3_Al and Ni_5_Al_3_ layers were 21 μm and 2 μm, respectively. Fine Ni_3_Al precipitates were observed in the Ni layers and showed a gradient distribution. As the hot pressing temperature rose to 1150 °C (Figure 4b), the average thickness of the Ni_3_Al layers gradually decreased to 6 μm, and the Ni_5_Al_3_ layers were undetected. The average size of the Ni_3_Al precipitates in Ni layers gradually increased with the temperature. When the hot pressing temperature rose to 1200 °C, the laminated structure disappeared (Figure 4c). The results suggested that the coarse Ni_3_Al precipitates were homogeneously distributed in the Ni layers. The average size of the Ni_3_Al precipitates was about 200 µm.

**Figure 5:**
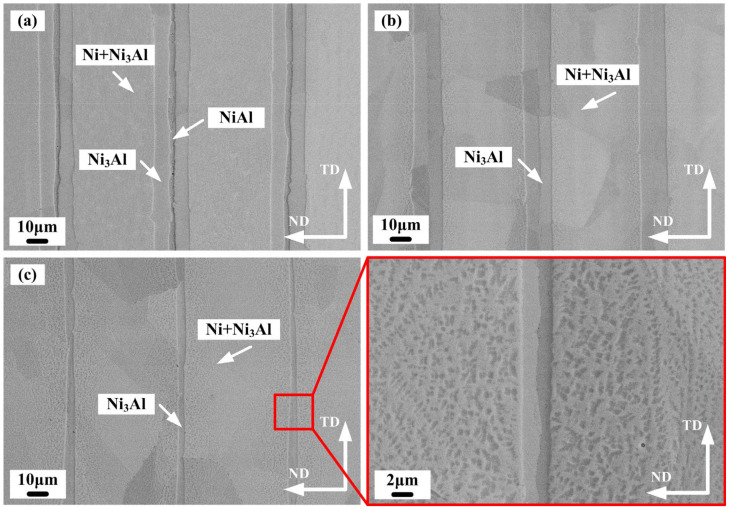
BSE images of the laminated composites: (**a**) N4; (**b**) N5; (**c**) N2.

Figure 5 shows the cross-section BSE image of multilayer composites fabricated under various holding times. It can be seen from Figure 5a that the microstructure was mainly composed of Ni, NiAl, and Ni_3_Al phases based on our EDS results. The thickness of the Ni layer was the largest, and a NiAl layer was observed in the middle of the Ni_3_Al layer with a continuous distribution. The NiAl and Ni_3_Al intermetallics were also observed after reaction under heat treatment at high temperature by Kim et al. [7]. The average thicknesses of the Ni_3_Al and NiAl layers were 21 μm and 4.2 μm, respectively. The Ni_3_Al layer was adjacent to the Ni layer, followed by the NiAl layer. This sequential phase distribution was consistent with the experimental results of our previous study in the high-temperature reaction synthesis stage, suggesting a certain dependence between the Ni and Ni_3_Al layers during the reaction. The Ni_3_Al phase formed at the interface between the Ni and NiAl phases, and the Ni_3_Al precipitates formed in the Ni phase. The Ni_3_Al phase formed in the Ni phase in the following orientation: {100}_Ni3Al_∣{100}_Ni_ and <001>_Ni3Al_∣<001>_Ni_. This resulted in a flat bond between the Ni and Ni_3_Al layers [12]. The Ni_3_Al matrix was larger closer to the Ni_3_Al layer, which contributed to grain boundary diffusion [12]. As the holding time increased to 1 h, the NiAl layer disappeared, and the microstructure only consisted of Ni and Ni_3_Al layers. The average thickness of the Ni_3_Al layer decreased to 18.3 μm, as shown in Figure 5b. When the holding time increased to 2 h, the average thickness of the Ni_3_Al layer decreased to 6 μm. The closer to the Ni_3_Al layer, the larger the size and volume fraction of Ni_3_Al precipitates, as shown in Figure 5c.

### 3.3. Mechanical Properties of the Laminated Composites

**Figure 6:**
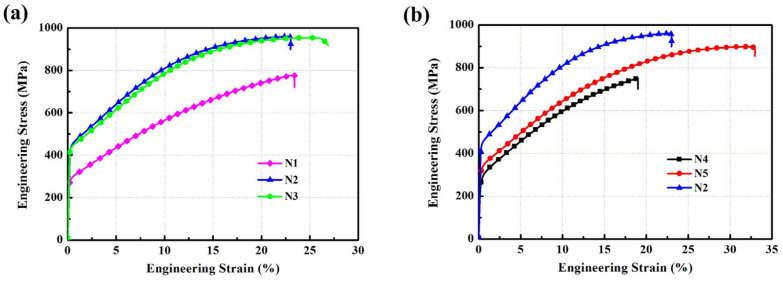
Tensile stress–strain curves of the laminated composites at room temperature for: (**a**) N1, N2, and N3; (**b**) N4, N5, and N2.

The typical tensile stress–strain curves of the laminated composites under different hot pressing conditions at room temperature with a strain rate of 1 × 10^−3^ s^−1^ are shown in Figure 6. The yield strength, ultimate tensile strength, and elongation are listed in Table 3. The yield strength and ultimate tensile strength of the Ni foils with a thickness of 0.06 mm at room temperature were 119.8 MPa and 345.9 MPa, respectively [22]. Additionally, the yield strength and ultimate tensile strength of the Al foils with a thickness of 0.1 mm were 35.6 MPa and 66.8 MPa, respectively [22]. It can be seen that the tensile strength values of the laminated composites were significantly improved compared with the Ni foils and Al foils.

Figure 6a displays the tensile stress–strain curves of the laminated composites (marked N1, N2, and N3) fabricated under 620 °C/5 MPa/1 h + 1100–1200 °C/10 MPa/2 h. As the hot pressing temperature increased from 1100 °C to 1150 °C, the yield strength increased from 290 MPa to 443 MPa, and the ultimate tensile strength increased from 778 MPa to 965 MPa. However, the yield strength and ultimate tensile strength did not change significantly when the hot pressing temperature further increased from 1150 °C to 1200 °C. Figure 6b displays the tensile strength of the laminated composites (marked N4, N5, and N2) processed under 620 °C/5 MPa/1 h + 1150 °C/10 MPa/0.5–2 h. When the holding time increased from 0.5 h to 1 h, the yield strength increased from 281 MPa to 319 MPa, and the ultimate tensile strength increased from 748 MPa to 887 MPa. When the holding time was increased to 2 h, the yield strength increased to 443 MPa and the tensile strength increased to 965 MPa. It can be seen that the Ni_3_Al layers enhanced the room-temperature mechanical properties of the laminated materials. However, improvement in the room-temperature strength of the laminated composites was limited by the continued addition of discontinuously distributed Ni_5_Al_3_ layers and continuously distributed NiAl layers in the Ni_3_Al layers. In addition, Ni_3_Al existed as a strengthening phase, mainly in the form of dispersed particles and layers. The effects of these two forms on the strength of the laminated composites were similar when the total content of the Ni_3_Al reinforcing phase was constant. However, the effect on elongation enhancement was greater when Ni_3_Al was present in the form of a lamellar reinforcing phase. The greater thickness of the Ni_3_Al layers contributed to the strength of the laminated composites but reduced the elongation.

**Figure 7:**
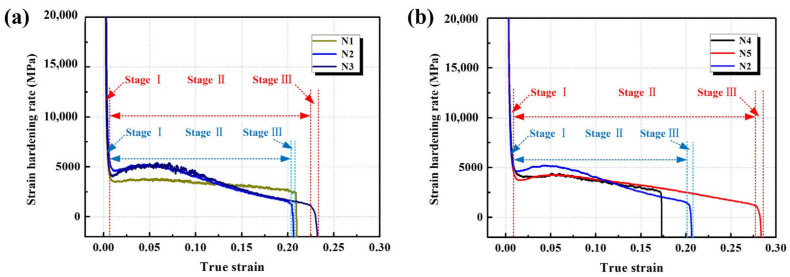
Work hardening rate curves of the sheet at room temperature for (**a**) N1, N2, and N3; (**b**) N4, N5, and N2.

Based on the previous room-temperature tensile tests, the laminated composite sheets exhibited an excellent combination of strength and toughness. The laminated composites prepared under different hot-pressing parameters exhibited different work-hardening abilities during deformation. It was essentially the variability of the microstructure that affected its strength and plasticity [23]. The work-hardening curves are an essential method for statistically evaluating the work-hardening behavior. Figure 7a depicts the plotted work hardening rate vs. true strain for materials fabricated under different processes. The work hardening rate was calculated by $\Theta ={\left(\partial \sigma /\partial \varepsilon \right)}_{\dot{\varepsilon }}$. In the linear hardening stage (Stage Ι), the strain hardening rate dropped linearly upon increasing the strain for all samples, and the strain hardening rate curves overlapped. Then, the curve of sample N1 dropped linearly during the homogenous hardening stage (Stage II). In this stage, the peaks of the curves for samples N2 and N3 appeared at a strain of 0.05, followed by a rapid decline to necking instability. In contrast to samples N2 and N3, samples N4 and N5 exhibited comparable tendencies, with a strain peak at 0.06, followed by a subsequent decline. The strain hardening rate of samples N2 and N3 was higher than that of the samples N4 and N5 (Figure 7b), indicating the presence of extra strain hardening. The interaction and mutual entanglement of dislocations led to a rapid proliferation of dislocations, which was manifested by a small increase in the strain hardening rate. The increase in samples N2 and N3 was significantly greater than that of N4 and N5. It can be seen that the morphology, size, and distribution of the intermetallic compound reinforcement phases significantly impacted the strain hardening rate of the layered materials. The contribution of the layered NiAl phase was lower than that of the layered Ni_3_Al phase. Both the laminated and precipitated Ni_3_Al phases increased the work hardening rates of the laminated composites, resulting in an enhanced capacity to form, drag, and pin dislocations. The higher strength and ductility were thought to be the result of heterogeneous deformation-induced strengthening and strain hardening.

**Figure 8:**
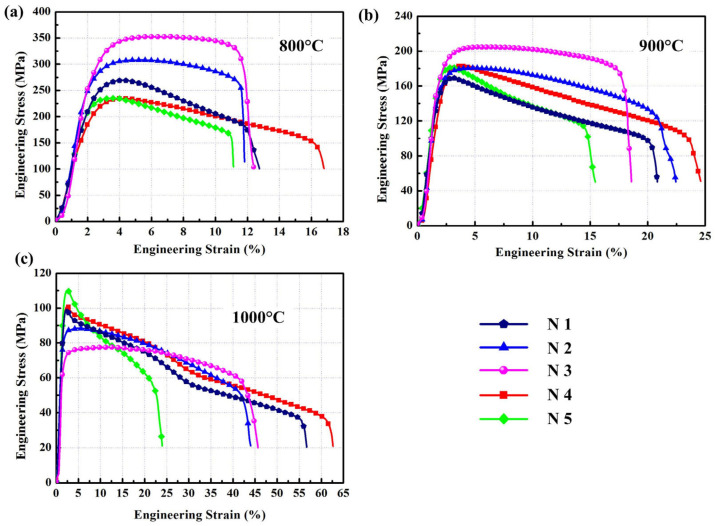
Tensile stress–strain curves of the laminated composites at elevated temperatures: (**a**) 800 °C; (**b**) 900 °C; (**c**) 1000 °C.

Figure 8 shows the tensile stress–strain curves of the laminated composites obtained under different hot pressing parameters tested at elevated temperatures. The yield strength, ultimate tensile strength, and elongation of the laminated composites at temperatures ranging from 800 °C to 1000 °C and strain rate of 1 × 10^−3^ s^−1^ are listed in Table 4. The thickness of the NiAl and Ni_3_Al layers significantly affected the high-temperature tensile strength of the laminated composites. The thickness of the intermetallic compound layer was positively correlated with the strength of the laminated composites at 1000 °C. The effect of the Ni_3_Al layer on improving the high-temperature strength of the laminated composites was greater than that of the NiAl layer, but the Ni_3_Al layer did not improve the elongation. The results showed that the dispersed Ni_3_Al phase had a greater effect on the strength of the laminated composites than when in the form of a layered structure. When the Ni_3_Al phase was distributed in a laminar structure, NiAl and Ni_3_Al were thinner, which resulted in a higher strength and greater elongation of the laminated composites.

### 3.4. Fracture Characteristics of the Laminated Composites

**Figure 9:**
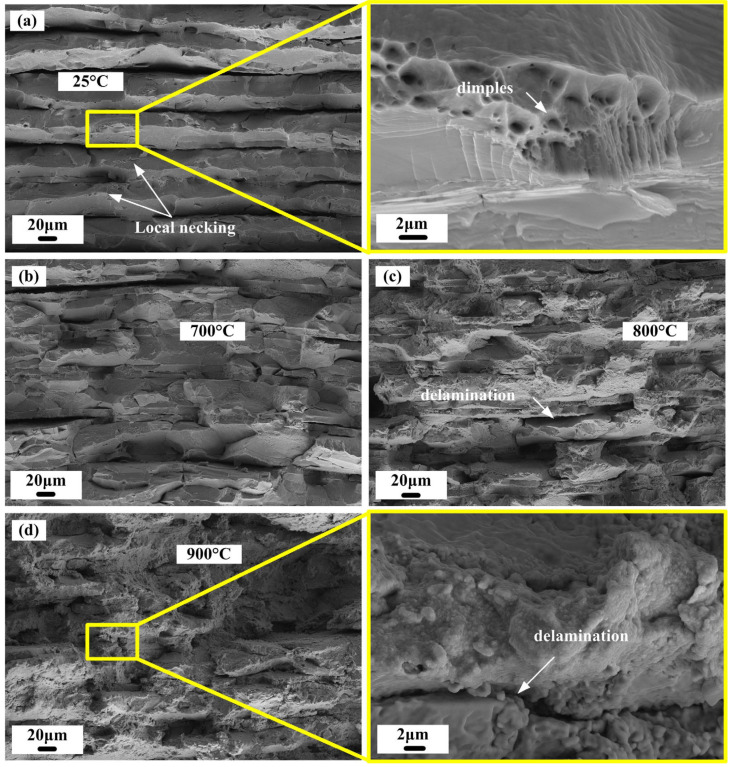
Fracture morphology of the tensile specimens at (**a**) 25 °C; (**b**) 700 °C; (**c**) 800 °C; (**d**) 900 °C.

Figure 9 depicts the fracture morphologies after uniaxial tensile tests at different temperatures. The sample was fabricated under 620 °C/5 MPa/1 h + 1100 °C/10 MPa/2 h. As shown in Figure 9a, plastic and intermetallic compound layers were clearly distributed at the fracture sites of the specimens at room temperature. The fracture surface was relatively rough and revealed noticeable grooves, and the specimens failed in a jagged fracture mode along the loading direction. Many dimples were present at the fracture site of the Ni layers, and the height of the failed Ni layers was greater than that in the intermetallic compound layers, which indicated that final local ductile failure occurred in the Ni layer. In addition, clear delamination was observed between the Ni layer and the intermetallic compound layer, indicating that the dislocation density at the interface exceeded the bonding strength of the interface. The release of local stress was achieved through debonding. Figure 9b–d show that the intermetallic layers cracked parallel to the direction of the applied force under tensile stress at higher temperatures. The intermetallic layers experienced delamination only near the fracture surface, and nickel layers underwent ductile fracture. The morphology of the high-temperature tensile specimen fractured at 700–800 °C was significantly different from that of the room-temperature fracture. The depth of the grooves on the fracture surface was significantly lower than that of the room-temperature fracture. Upon increasing the temperature, the interface between the Ni plastic layer and the intermetallic compound layer at the fracture surface was gradually blurred, and delamination tended to be weaker. This indicated that the plastic deformation capacity of both the Ni layer and the intermetallic compound layer was improved at high temperatures. The improved synergistic deformation ability was accompanied by the mitigation of local stress concentration at the interface.

The fracture behavior of the laminated composites can be explained using the classical cantilever beam mechanics model [24], as follows:

(1) $$\sigma =\frac{10}{11}\cdot \frac{\delta \cdot E\cdot t_{IMCs}^{3}}{L^{4}}$$

where *σ* is out-of-plane stress perpendicular to the thickness direction between Ni and the intermetallic layers (IMCs), *δ* is the deflection of the intermetallic layers, *t_IMCs_* is the thickness of the intermetallic layers, *L* is the length of the cantilever beam, and *E* is the Young’s modulus of the intermetallic layers.

The interlayer normal stress was positively correlated with the deflection at the fracture of the IMCs for the same strain at fracture. The deformation temperature was negatively correlated with the deflection at the fracture of the IMCs. Therefore, decreasing the temperature increased the normal stress between the layers; the increase in temperature, in turn, decreased the normal stress between the layers. *δ*/*σ* can be used to represent the confinement effect between the interface of the Ni layer and the IMCs. Thus, it can be concluded that *δ*/*σ* was positively correlated with the strain at fracture, i.e., the larger the strain, the stronger the interfacial confinement effect on the material. The interlayer confinement was enhanced at low strain rates or high temperatures. Conversely, the interlayer confinement effect was weakened.

### 3.5. Interfacial Evolution during Hot Tensile Deformation

**Figure 10:**
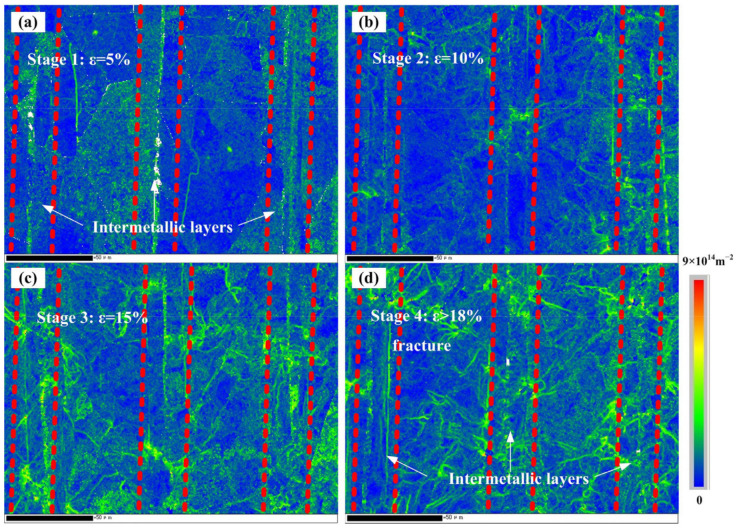
GND density maps of laminated composites after tensile deformation at 900 °C and 0.001/s with true strain of: (**a**) 5%; (**b**) 10%; (**c**) 15%; (**d**) fracture (>18%).

During hot tensile deformation, the interface and each group element shared the load transfer, strain distribution, and control during plastic deformation. The interface played an important role in coordinating the deformation among the group members. The inhomogeneous strain distribution was often related to the accumulation and stacking of geometrically necessary dislocations (GNDs) during plastic deformation. Therefore, GNDs in Ni and Ni_3_Al layers and their evolution trends can be obtained by quantitative calculations of the EBSD data. The kernel average misorientation (KAM) method can be used to calculate the local misorientation from the EBSD data to indirectly obtain the deformation laws under the limiting effect of the laminate materials. Misorientations greater than 3° were not taken into account during the calculation of local misorientation to exclude subgrain boundaries. The local misorientation is defined as the average misorientation between a central point (0.1 × 0.1 μm^2^) and its eight surrounding points:

(2) $$\theta_{local}=\sum_{i=1}^{8}\theta_{i}\cdot I_{\left(\theta_{i}<\alpha \right)}/\sum_{i=1}^{8}I_{\left(\theta_{i}<\alpha \right)}$$

where *θ_local_* is the calculated local misorientation for the corresponding point, and *θ_i_* is the misorientation between point *i* and its surrounding points; $I_{\left(\theta_{i}<\alpha \right)}$ is an indicator function, and *α* is the misorientation threshold (3 here). The GND density of a pixel (*ρ_GND_*) is presented by the Kubin strain gradient theory [25]:

(3) $$\rho_{GND}=\frac{2\theta }{ub}$$

where *ρ_GND_* is the GND density, *θ* is the local misorientation, *u* is the unit length (100 nm here), and *b* is the Burger vector (0.253 nm for face-centered cubic, and 0.248 nm for body-centered cubic). Figure 10 shows the GND density maps of the laminated composites at different tensile strains hot pressed under 620 °C/5 MPa/1 h + 1100 °C/10 MPa/2 h. Overall, the density of the GND increased significantly upon increasing the tensile strain. This was a typical deformation phenomenon and was the same as the results reported in a previous study [25]. The GND density of the Ni layers increased from 4.19 × 10^14^ m^−2^ to 5.74 × 10^14^ m^−2^ after deformation. The GND density of the intermetallic layers increased from the original 4.81 × 10^14^ m^−2^ to 6.48 × 10^14^ m^−2^, with higher values than that of the Ni layer. However, the GND density distribution was non-uniform and showed a regular gradient distribution. The GND density in the Ni layer was significantly lower than that in the intermetallic layer. The non-homogeneity specific to the material organization inside, such as grain boundaries, phase interfaces, and crystal orientation, resulted in an inhomogeneous GND density during plastic deformation. The GND density was closely related to the strain gradient, indicating that the intermetallic layers needed to adapt to the overall deformation by more GND in the form of dislocation stacking when the laminate material was deformed in concert.

## 4. Discussion

### 4.1. Reaction Synthesis Mechanism of the Laminated Composites

The reaction synthesis between Ni and Al foils was highly dependent on the diffusion of Ni and Al atoms on surfaces. According to the Ni-Al binary phase diagram, the laminated Ni/Ni_3_Al sheets were a multilayered system that included a series of Ni-Al intermetallic compounds, including NiAl_3_, Ni_2_Al_3_, NiAl, and Ni_3_Al. Intermetallic growth, transition, and stabilization were the three vital stages of intermetallic layer generation. According to previous reports [26,27,28] and our studies [22,29], the synthesis from step 1 to step 4 is illustrated below:

(4) $$\mathrm{Stage}\;1:\;\mathrm{Ni}+\mathrm{Al}\to {\mathrm{NiAl}}_{3}+\Delta H_{1}$$

(5) $$\mathrm{Stage}\;2:\;{\mathrm{NiAl}}_{3}+\mathrm{Ni}\to {\mathrm{Ni}}_{2}{\mathrm{Al}}_{3}+\Delta H_{2}$$

(6) $$\mathrm{Stage}\;3:\;{\mathrm{Ni}}_{2}{\mathrm{Al}}_{3}+\mathrm{Ni}\to \mathrm{NiAl}+\Delta H_{3}$$

(7) $$\mathrm{Stage}\;4:\;\mathrm{NiAl}+\mathrm{Ni}\to {\mathrm{Ni}}_{3}\mathrm{Al}+\Delta H_{4}$$

**Figure 11:**
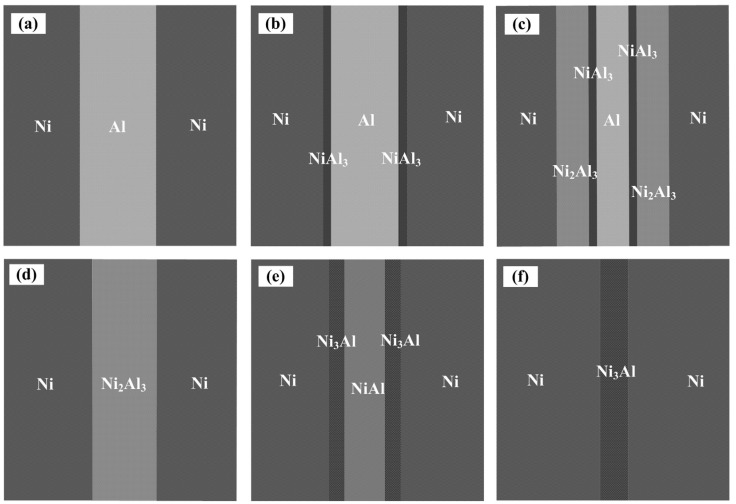
Schematic illustration of the reaction synthesis mechanism of the laminated composites. (**a**) The hot-pressed Ni/Al/Ni laminated composites; (**b**) NiAl_3_ layers are formed between Ni and Al layers; (**c**) Ni_2_Al_3_ layers are formed between NiAl_3_ and Ni layers; (**d**) NiAl_3_ disappears and forms the Ni/Ni_2_Al_3_/Ni lamellar structure; (**e**) Ni_3_Al layers are formed between NiAl and Ni layers; (**f**) NiAl disappears and forms the Ni/Ni_3_Al/Ni lamellar structure.

Figure 11 displays a schematic illustration of the reaction synthesis mechanism of the laminated composites. The temperature-induced reaction synthesis can be divided into four steps, ranging from room temperature to 1150 °C. In the first stage, the NiAl_3_ layer emerged at the Ni/Al interface and then developed continuously along the thickness direction. In this stage, the diffusion reaction occurred rapidly, followed by the second stage. NiAl_3_ was an intermediate product in this process, but the NiAl_3_ and Ni_2_Al_3_ phases usually coexisted. Ni/Al underwent a long-term solid-state reaction synthesis at a constant temperature of 620 °C. In this stage, the Al layer provided sufficient Al atoms for the diffusion reaction of the NiAl_3_ and Ni_2_Al_3_ phases, resulting in a continual increase in the thickness of the NiAl_3_ and Ni_2_Al_3_ layers. However, as the thickness of the Al layer gradually diminished, the supply of Al atoms also gradually diminished, resulting in the development and expansion of Ni_2_Al_3_, which depended even more on the NiAl_3_ layer. When the reaction synthesis temperature reached 854 °C, a small amount of a liquid–solid mixed phase formed at the phase boundary between NiAl_3_ and Ni_2_Al_3_, leading to an intensification of the reaction. The NiAl_3_ layer subsequently disappeared and developed into a Ni/Ni_2_Al_3_/Ni lamellar structure. Upon increasing the temperature, the NiAl phase gradually generated and occupied the position of Ni_2_Al_3_ phase. When the temperature increased to 1133 °C, a solid–liquid mixture appeared in the reaction zone. At this time, the decomposition efficiency and reaction rate of the remaining Ni_2_Al_3_ phase reached an extreme value. In the third and fourth stages, as the NiAl phase was generated, the Ni_3_Al phase formed at the Ni/NiAl phase boundary, which was related to the controlled induction of grain boundary diffusion. As the diffusion time increased, the thickness of the NiAl phase gradually decreased until it finally disappeared, and a Ni/Ni_3_Al/Ni lamellar structure formed. The Ni_3_Al phase existed mainly as a lamellar structure or in the form of precipitates in the Ni matrix. As the diffusion time continued to increase, the thickness of the Ni_3_Al layer gradually decreased until it finally disappeared. However, the volume fraction of Ni_3_Al precipitates in the Ni matrix showed a gradient distribution in the thickness direction. When the diffusion time was sufficient, the volume fraction of Ni_3_Al precipitates in the Ni matrix was gradually homogenized.

### 4.2. Mechanical Response of the Laminated Structure

The layer –thickness relationship of the laminated structure and its matching pattern were important factors affecting the strength and elongation of the laminated composites. The strength and strain-hardening capacity of the Ni and Ni_3_Al layers were very different. As a result, the laminated composites displayed significant heterogeneity during overall deformation, in contrast to the homogeneous material. During the deformation process, the Ni layer first yielded, and the Ni_3_Al layer underwent elastic deformation. The deformation of the Ni layer was restricted by the Ni_3_Al layer. When sufficient interfacial bonding was maintained between the Ni and Ni_3_Al layers, the laminated material underwent simultaneous plastic deformation, and a large strain gradient appeared near the interface between the Ni and Ni_3_Al layers. This was fully verified by the experimental results shown in Figure 10. At the initial stage of deformation, significant dislocation density accumulation was observed near the intermetallic compound layer and the interface region, demonstrating the storage of GNDs in the heterogeneous interface region. Upon increasing the plastic strain, the inhomogeneous distribution of GNDs at the layer interface was obvious, and the heterogeneous structure led to a complex stress state transmitted by the additional stresses near the interface. This contributed to dislocation propagation and entanglement, and the stresses began to gradually expand toward the Ni layer along the thickness direction. The GNDs in the Ni layer originated from the generation of their own plastic deformation and accumulation when accommodating the strain gradient, according to the strain gradient plasticity theory [25].

**Figure 12:**
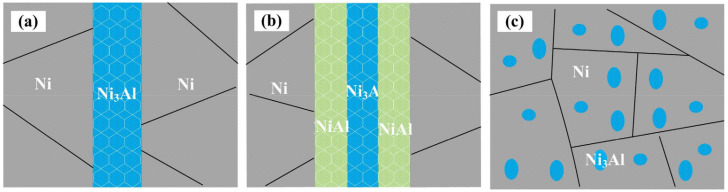
Microstructure schematics of different phase structure forms. (**a**) Dual-phase with Ni matrix layer phases that contains Ni_3_Al layers; (**b**) ternary-phase with Ni matrix layers phase that contains Ni_3_Al and NiAl layers; (**c**) dual-phase with Ni matrix phase that contains Ni_3_Al particles.

The average thicknesses of the Ni_3_Al layers in samples N2 and N5 were about 6 μm and 18.3 μm, respectively. The average thicknesses of the Ni layers in samples N2 and N5 were about 65.4 μm and 55.2 μm, respectively. The stress of the laminates was influenced by the thickness of each layers according to the rule of mixtures [30,31].

(8) $$\sigma_{m}=V_{i}\sigma_{i}+(1-V_{i})\sigma_{\mathrm{Ni}}$$

(9) $$V_{i}=\frac{t_{i}}{t_{i}+t_{\mathrm{Ni}}}$$

where *σ_m_* is the fracture strength of the material, *σ*_Ni_ is the fracture strength of the Ni layer, *σ_i_* is the fracture strength of the Ni_3_Al intermetallic layer, *V_i_* is the volume fraction of the Ni_3_Al intermetallic, *t_i_* and *t*_Ni_ refer to the thicknesses of the Ni_3_Al intermetallic layer and Ni layer, respectively, measured by SEM. Therefore, it is very important to control the microstructure and thickness of the obtained Ni_3_Al layers. In samples N2 and N5, the thickness of the Ni layer increased, and the thickness of the Ni_3_Al layer decreased in the non-homogeneous laminated material. GNDs were accommodated more fully in the Ni layer, producing a significant hetero-deformation-induced strengthening and hardening effect, which in turn increased the yield strength at room temperature. This enhancement continued until near 900 °C. At higher temperatures (1000 °C), the hetero-deformation-induced strengthening and hardening effect of GNDs in the Ni layer decreased, while the high-strength effect of Ni_3_Al was fully manifested. Thus, the desired strengthening effect was not achieved. This conclusion was confirmed by experiments (combined with samples N2 and N4). In other words, the material’s strength could not be enhanced by simply adding intermetallic compound phase layers, which required the design of matching models for the Ni_3_Al, NiAl, and Ni layers. The structural form of the Ni_3_Al phase had a significant effect on the strength and plasticity of the stacked material, as illustrated by combining samples N2 and N3. The laminated Ni_3_Al phase had a significant high-temperature strengthening effect compared with the uniform distribution in the Ni matrix, but it could not achieve a high strength effect at room temperature or a low temperature. Figure 12 shows the microstructure schematic of different phase structures. The insight gained from this experimental phenomenon was that the reinforcement form of the material should not be limited to a lamellar structure but should be enhanced by adding different reinforcement effects to achieve overall strengthening at all temperature intervals. This, in turn, provided a reference for the preparation of high-performance metal–intermetallic compound materials.

## 5. Conclusions

The Ni/Ni_3_Al laminated composites were successfully fabricated by hot pressing with 0.6 mm Ni and 0.02 mm Al foils through an in situ solid-state reaction synthesis. The main conclusions are summarized as follows:

(1) The evolution of the interface phases in the Ni/Ni_3_Al laminated composite was shown to be NiAl_3_, Ni_2_Al_3_, NiAl, and Ni_3_Al in sequence. An excellent interface between the Ni and Ni_3_Al layers without cracks and voids was observed due to the uniform pressure applied during hot pressing.
(2) The laminated composites hot pressed under 620 °C/5 MPa/1 h + 1150 °C/10 MPa/2 h exhibited a better ultimate tensile strength of 965 MPa and an elongation of 22.6% at room temperature, accompanied with the high-temperature tensile properties with an ultimate tensile strength of 89 MPa and an elongation of 43.8% at 1000 °C.
(3) The layer–thickness relationship of the laminated structure and its matching pattern were important factors affecting the strength and elongation of the laminated composites over a wide range of temperatures, i.e., the alternating Ni_3_Al and the Ni matrix layers, as well as the dispersed Ni_3_Al precipitate phases.

Future work will elucidate the mechanical response mechanism of different phase structures with quantitative index and establish the correspondence relationship between Ni/Al foil thickness ratios and mechanical properties.

## Figures and Tables

**Table 1:** The hot pressing processing parameters for the laminated Ni/Al foils and their corresponding marks.

Marks	Parameters
N1	620 °C/5 MPa/1 h + 1100 °C/10 MPa/2 h
N2	620 °C/5 MPa/1 h + 1150 °C/10 MPa/2 h
N3	620 °C/5 MPa/1 h + 1200 °C/10 MPa/2 h
N4	620 °C/5 MPa/1 h + 1150 °C/10 MPa/0.5 h
N5	620 °C/5 MPa/1 h + 1150 °C/10 MPa/1 h

**Table 2:** Compositions of reacted Ni-Al system products determined by EDS in Figure 3.

Position	Average Chemical Composition/at. %		Phase Identity
	Ni	Al	
1	77.3	22.8	Ni_3_Al
2	77.6	22.4	Ni_3_Al
3	83.0	17.0	Ni + Ni_3_Al (Precipitates)

**Table 3:** Tensile properties of the laminated composites at room temperature.

Sample	Yield Strength/MPa	Ultimate Tensile Strength/MPa	Elongation/%
N1	290	778	22.7
N2	443	965	22.6
N3	450	960	27.2
N4	281	748	18.5
N5	319	887	36.5

**Table 4:** Tensile properties of the laminated composites at elevated temperatures.

Sample	Temperature/°C	Yield Strength/MPa	Ultimate Tensile Strength/MPa	Elongation/%
N1	800	216	269	12.8
	900	145	169	20.8
	1000	94	99	56.6
N2	800	248	307	11.8
	900	142	181	22.4
	1000	76	89	43.8
N3	800	263	352	12.4
	900	156	204	18.6
	1000	64	77	45.6
N4	800	192	234	16.8
	900	137	183	24.6
	1000	92	101	62.6
N5	800	203	235	11.1
	900	158	181	15.4
	1000	99	110	23.9

## Data Availability

The data presented in this study are available upon request from the corresponding author.
